# Combined targeting of Arf1 and Ras potentiates anticancer activity for prostate cancer therapeutics

**DOI:** 10.1186/s13046-017-0583-4

**Published:** 2017-08-23

**Authors:** Liwei Lang, Chloe Shay, Xiangdong Zhao, Yong Teng

**Affiliations:** 1 Department of Oral Biology, Augusta University, Augusta, GA 30912 USA; 2 Department of Pediatrics, Emory Children’s Center, Emory University, Atlanta, GA 30322 USA; 3 Georgia Cancer Center, Augusta University, 1120 15th Street, Augusta, GA 30912 USA; 4 Department of Biochemistry and Molecular Biology, Augusta University, Augusta, GA 30912 USA

**Keywords:** Prostate cancer, Arf1, Ras, Exo2, Salirasib, Combination treatment

## Abstract

**Background:**

Although major improvements have been made in surgical management, chemotherapeutic, and radiotherapeutic of prostate cancer, many prostate cancers remain refractory to treatment with standard agents. Therefore, the identification of new molecular targets in cancer progression and development of novel therapeutic strategies to target them are very necessary for achieving better survival for patients with prostate cancer. Activation of small GTPases such as Ras and Arf1 is a critical component of the signaling pathways for most of the receptors shown to be upregulated in advanced prostate cancer.

**Methods:**

The drug effects on cell proliferation were measured by CellTiter 96® AQueous One Solution Cell Proliferation Assay. The drug effects on cell migration and invasion were determined by Radius™ 24-well and Matrigel-coated Boyden chambers. The drug effects on apoptosis were assessed by FITC Annexin V Apoptosis Detection Kit with 7-AAD and Western blot with antibodies against cleaved PARP and Caspase 3. A NOD/SCID mouse model generated by subcutaneous injection was used to assess the in vivo drug efficacy in tumor growth. ERK activation and tumor cell proliferation in xenografts were examined by immunohistochemistry.

**Results:**

We show that Exo2, a small-molecule inhibitor that reduces Arf1 activation, effectively suppresses prostate cancer cell proliferation by blocking ERK1/2 activation. Exo2 also has other effects, inhibiting migration and invasion of PCa cells and inducing apoptosis. The Ras inhibitor salirasib augments Exo2-induced cytotoxicity in prostate cancer cells partially by enhancing the suppression of ERK1/2 phosphorylation. In a xenograft mouse model of prostate cancer, Exo2 reduces prostate tumor burden and inhibits ERK1/2 activation at a dose of 20 mg/kg. Synergistic treatment of salirasib and Exo2 exhibits a superior inhibitory effect on prostate tumor growth compared with either drug alone, which may be attributed to the more efficient inhibition of ERK1/2 phosphorylation.

**Conclusion:**

This study suggests that simultaneous blockade of Arf1 and Ras activation in prostate cancer cells is a potential targeted therapeutic strategy for preventing prostate cancer development.

**Electronic supplementary material:**

The online version of this article (doi:10.1186/s13046-017-0583-4) contains supplementary material, which is available to authorized users.

## Background

Prostate cancer is the second most common cause of cancer-related deaths despite advances in screening and treatment over the past decade [1]. Various treatment options for prostate cancer, including hormone deprivation and chemotherapy, largely depend on the severity of disease, functional status, age and genetic background (e.g. androgen receptor activity). Androgen deprivation therapy (ADT) is the first line therapy for locally advanced or metastatic prostate cancer; however it is associated with significant adverse effects (e.g. osteoporosis, fatigue, and fatal cardiac events) and inevitably becomes ineffective once the cancer progresses to metastatic castration-resistant prostate cancer (mCRPC) [2, 3]. Current monotherapies, particularly with antiandrogens, are less effective and only exhibit activity in limited clinical settings, which may be attributed to the intrinsic and complex heterogeneity of prostate cancer [3, 4]. Chemotherapies are often used to treat prostate cancer that is resistant to hormone ablation therapy. A good example for chemotherapeutic treatment is docetaxel with prednisone, which has been shown to be effective in regression of metastatic hormone refractory Prostate cancer [5]. However, current chemotherapies are always associated with side effects which must be considered before finalizing the treatment strategy. Therefore, the identification of new central molecular targets in cancer progression and development of new targeted therapies or improved treatment regimens are very necessary for achieving better survival for patients with prostate cancer.

Several lines of evidence have shown that growth factors such as EGF and IGF-I are overexpressed in advanced prostate cancer, promoting tumorigenesis by activating two canonical cancer-driving mitogen-activated protein kinase (MAPK) and phosphoinositide-3-kinase (PI3K) pathways [6, 7]. Oncogenic Ras has been implicated in the most fatal cancers and functions as an intersection point to link diverse growth factors to MAPK/PI3K pathways [8, 9]. Unlike other types of malignancy, oncogenic Ras mutations are infrequent in prostate cancer patients; however wild-type Ras can be chronically activated by autocrine and paracrine growth factor stimulation in prostate cancer [10, 11]. Therefore, blockade of MAPK/PI3K signaling cascades by inhibiting Ras activity represents a potential modality for therapeutic intervention of prostate cancer.

Ras is considered as “undruggable” because it lacks a well-defined binding pocket in the Ras protein structure to accommodate a biologically active small molecule [12, 13]. As research on drugs targeting oncogenic Ras directly was unsuccessful, the focus for developing anti-Ras cancer drugs has shifted to the modulation of proteins involved in Ras activation. The Ras antagonist salirasib, also known as farnesylthiosalicylate (FTS), can bind to the Ras membrane binding site and dislodge GTP-bound Ras from its membrane anchorage domain, which ultimately accelerates degradation of the GTP-bound Ras in the cytoplasm [14–16]. Salirasib exhibits anticancer effects in many cancer cell lines, at least partially mediated by reducing MAPK activity [17–19]. Given the fact that salirasib antagonizes Ras activity specifically by competing with the active GTP-bound form, it may be a more specific means of treating advanced prostate cancer.

ADP-ribosylation factors (Arfs) are a family of Ras-related GTP binding proteins, which are well characterized as important regulators for vesicular trafficking [20, 21]. Out of the Arfs, Arf6 in particular has received much attention in the past several years. Arf6 is linked to cancer invasion and metastasis since its functions are associated with actin cytoskeletal remodeling, cell polarity and migration [22]. Arf6 expression is significantly elevated in prostate cancer clinical samples and it regulates ErbB3 nuclear localization in prostate cancer cells [23, 24]. Like Arf6, Arf1 acts as a molecular switch in cellular signaling by cycling between GTP-bound active and GDP-bound inactive states. Arf1 activity is precisely controlled by Arf1-directed guanine nucleotide exchange factors (Arf1 GEFs) and GTPase-activating proteins (Arf1 GAPs) [20]. Our previous study has demonstrated that elevated levels of Arf1 in prostate cancer cells positively correlate with hyperactivation of the ERK1/2 MAPK pathway [25]. Depletion of Arf1 in prostate cancer cells impairs ERK1/2 activation and suppresses cell proliferation in vitro and prostate tumorigenesis in vivo [25]. Most commercially available Arf1 inhibitors, including Brefeldin A (BFA) and Secin H3, block its activation by targeting the Sec7 domain on Arf1-GEFs [26, 27]. In contrast to this class of Arf1 inhibitors, Exo2 interferes with the function of Arf1 or Arf1-GEFs localized to the ER-Golgi intermediate compartment or the trans-Golgi network [27]. Although Arf1 inhibitors can be used for assessing Arf1 function by inactivating a subset of Arf1-GEFs, their potential usage, particularly their in vivo anticancer efficacy and safety, remains to be established.

The aims of the present study are to evaluate the effects of Exo2 and salirasib in human prostate cancer cells and animal models of prostate tumors, and to understand its combination treatment efficacy and the underlying molecular mechanisms of synergistic action. This study suggests that combination of these two inhibitors may provide a more effective therapeutic option for patients with advanced prostate cancer.

## Methods

### Cell lines

Cancer cells PC3, DU145, 22Rv1, LNCaP, MDA-MB-231, T47D, H1299 and SW60 were obtained from American Type Culture Collection (ATCC) and passage <5 were used in this study. All cells were maintained in Dulbecco’s modified Eagle’s medium (DMEM) containing 10% fetal bovine serum.

### Reagents, antibodies and standard assays

Exo2, BFA and Secin H3 were obtained from Selleckchem (Houston, TX). Salirasib and β-actin antibody were purchased from Sigma-Aldrich (St Louis, MO). Antibodies that recognize p-AKT, p-ERK1/2, p-STAT3, p-Src, AKT, ERK1/2, STAT3, Src and cleaved (c)-PARP were purchased from Cell Signaling Technology (Beverly, MA). Ki67 and Arf1 antibodies were purchased from Abcam (Cambridge, MA). Cell proliferation was determined by CellTiter 96® AQueous One Solution Cell Proliferation Assay (MTS) (Promega, Madison, MI) and crystal violet staining. Apoptosis was determined by FITC Annexin V Apoptosis Detection Kit with 7-AAD (BioLegend, San Diego, CA). Western blot, Arf1 activation, and Transwell invasion analyses were carried out as described previously [21, 28–31].

### Gap closure migration assays

Cell migration was determined using the Radius™ 24-well from Cell Biolabs (San Diego, CA). In this assay, cells were seeded on Radius cell migration plates and allowed to form monolayers before circular gaps were generated by removing the gels. Cells were then treated with DMSO or different drugs for 24 h, and the migratory gaps were captured at the same magnification using a Zeiss LSM-510 inverted microscope (Zeiss, Germany).

### Animal models, drug administration and immunohistochemistry (IHC)

Six-week-old male NOD/SCID mice were purchased from the National Cancer Institute (NCI) and all animal experiments were approved by the Institutional Animal Care and Use Committee (IACUC) of Augusta University. To generate xenotransplantation models, exponentially growing PC3 cells (1.5 × 10^6^ cells) were suspended in 100 μl of PBS/matrigel (1:1) and injected subcutaneously into the right flanks of mice. One week after PC3 cell implantation, mice were randomized to receive control vehicle or drug(s) (*n* = 5). The control mice were injected intraperitoneally (*i.p.*) with 100 μl of sterile saline, whereas treatment groups received equal volume treatment of Exo2 and salirasib, alone or in combination. Exo2 or/and salirasib were administered intraperitoneally 5 days for beginning 1 week and every other day for the following 2 weeks. Tumor volume was measured externally every 3 to 5 days using vernier calipers as length × width^2^ × 0.52. The mice were sacrificed on treatment day 21, and the xenografts were removed and processed for IHC with Ki67 and p-ERK1/2 antibodies as described previously [25, 28, 31].

### Statistical analysis

Treatment effects were evaluated using a two-sided Student’s t test at each measurement time-point. To assess the longitudinal effect of treatment, a mixed model was employed to test the overall difference across all groups as well as between each pair of groups during the whole study period. The data were presented as means ± SD and *p* < 0.05 was considered significant.

## Results

### Exo2 inactivates ERK1/2 signaling and inhibits proliferation in prostate cancer cells

**Fig. 1:**
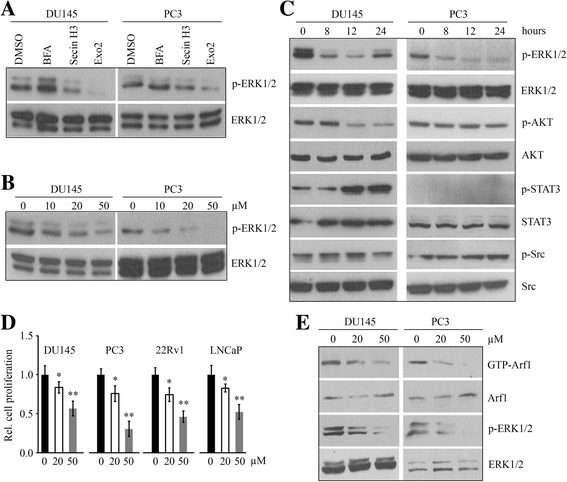
Exo2 blocks ERK1/2 activity and inhibits proliferation in prostate cancer cells. **a** DU145 and PC3 cells were treated with 20 μM of the indicated Arf1 inhibitors for 24 h, and cell lysates were collected for Western blot with the indicated antibodies. **b**, **c** DU145 and PC3 cells were treated with the indicated concentrations of Exo2 for 24 h (**b**) or 50 μM Exo2 for the indicated times (**c**), and cell lysates were collected for Western blot with the indicated antibodies. **d** Prostate cancer cell lines DU145, PC3, 22Rv1 and LNCaP were treated with the indicated concentrations of Exo2 for 72 h, and cell proliferation was determined by MTS. **e** DU145 and PC3 cells were treated with the indicated concentrations of Exo2 for 24 h, and GGA3-PBD agarose beads were used to pull down the GTP-bound Arf1 and Western blot was used to determine the indicated protein levels.*p < 0.05; **p < 0.01

Loss of Arf1 leads to reduced phosphorylation levels of ERK1/2 (25). Thus, we treated DU145 and PC3 cells with Arf1 inhibitors BFA, Secin H3 or Exo2 to explore whether they affect the MAPK pathway in prostate cancer cells. Neither BFA nor Secin H3 could reduce ERK1/2 phosphorylation significantly at 20 μM concentration, compared with the control (Fig. 1a). In contrast, Exo2 exhibited strong inhibitory effects on inactivation of ERK1/2 at the same concentration in both DU145 and PC3 cells (Fig. 1a). We next determined the effective dose of Exo2 by treating DU145 and PC3 cells with concentrations ranging from 10 μM to 50 μM. After 24 h of drug exposure, Western blot analysis showed that Exo2 effectively inactivated ERK1/2 at 20 μM and the inhibitory effect is dose-dependent (Fig. 1b). The time-course analysis of Exo2 treatment showed that phosphorylation of ERK1/2 was decreased following 8 h of exposure and much lower at 12 h after treatment (Fig. 1c). We further determined the possible pathway signaling involved in mediating the effects of Exo2 treatment. In the presence of Exo2, decreased phosphorylation of AKT was only found in DU145 cells, but not PC3 (Fig. 1c). Phosphorylation of STAT3 was not detectable in PC3 cells but it was increased in Exo2-treated DU145 cells (Fig. 1c). No significant changes in Src phosphorylation were observed with or without Exo2 (Fig. 1c). MTS assays showed that Exo2 inhibited proliferation of DU145, PC3 and another androgen-independent 22Rv1 cells in a dose-dependent manner (Fig. 1d). Different from DU145, PC3 and 22Rv1, LNCaP is androgen-dependent cells. Given the fact that AR signaling is also involved in the regulation of ERK1/2 signaling as well as prostate cancer survival, we next sought to determine the inhibitory effects of Exo2 on these cells. Similar tendency was observed in LNCaP cells that Exo2 dose-dependently suppressed cell proliferation. These data were confirmed by cell growth assays with crystal violet staining (Additional file 1: Figure S1), indicating that Exo2 has an anticancer activity in two major types of prostate cancer cells regardless of AR signaling. We next examined the effect of Exo2 on blocking Arf1 activation. The decreased GTP-bound Arf1 (activated form) was positively associated with reduced ERK1/2 activation (Fig. 1e). These data indicate that Exo2-induced repression of proliferation in prostate cancer cells, at least partially, through inhibiting the Arf1-ERK1/2 signaling cascade.

### Salirasib augments Exo2-induced repression of ERK1/2 activation and proliferation in prostate cancer cells

**Fig. 2:**
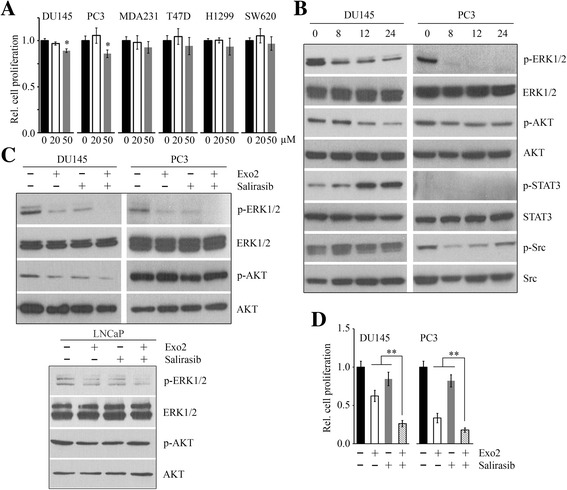
Salirasib enhances Exo2-induced repression of phospho-ERK1/2 and proliferation in prostate cancer cells. **a** Various cancer cell lines were treated with the indicated concentrations of salirasib for 72 h, and cell proliferation was determined by MTS assays. **b** DU145 and PC3 cells were treated with 50 μM salirasib for the indicated times, and cell lysates were collected for Western blot with the indicated antibodies. **c** DU145, PC3 and LNCaP cells were treated with 50 μM Exo2 and 50 μM salirasib for 24 h, alone or in combination, and cell lysates were collected for Western blot with the indicated antibodies. **d** DU145 and PC3 cells were treated with 50 μM Exo2 and 50 μM salirasib for 72 h, alone or in combination, and cell proliferation was determined by MTS assays. **p < 0.01

The Ras inhibitor salirasib can disrupt the spatiotemporal localization of active Ras through competing with Ras for binding to Ras-escort proteins [14–16, 32]. We treated various cancer cell lines with salirasib for 72 h and found no significant inhibitory effect on cell proliferation in the presence or absence of 20 μM salirasib (Fig. 2a). When the concentration was increased to 50 μM, salirasib inhibited proliferation of prostate cancer DU145 and PC3 cells, but not of other types of cancer cells (Fig. 2a). To study the possible mechanisms involved in salirasib-induced repression of cell proliferation, we determined multiple critical signaling pathways by Western blot which revealed decreased phosphorylation of ERK1/2 in both DU145 and PC3 cells (Fig. 2b). Similar to the effects of Exo2, decreased phosphorylation of AKT and increased phosphorylation of STAT3 were observed in salirasib-treated DU145 cells (Fig. 2b). A dramatic decrease in Src phosphorylation was detected in PC3 cells during the first 24 h after salirasib treatment, while there were no significant changes in phospho-Src levels in DU145 cells in the presence or absence of salirasib (Fig. 2b). We then evaluated the synergistic effect of Exo2 and salirasib in PC3 cells. This drug combination inactivated ERK1/2 and suppressed cell proliferation more efficiently than either drug alone (Fig. 2c and d). Addition of salirasib in the treatment of Exo2 augmented the inhibitory effect of Exo2 on AKT phosphorylation in DU145 cells, but not in PC3 and LNCaP cells (Fig. 2c), suggesting that ERK1/2 activation is a common downstream target of this combined treatment. These data support a notion that prostate cancer cell proliferation can be suppressed more efficiently by co-inhibition of Arf1- and Ras-mediated MAPK signaling.

### Combination of Exo2 and salirasib inhibits migration, invasion and apoptosis of prostate cancer cells

**Fig. 3:**
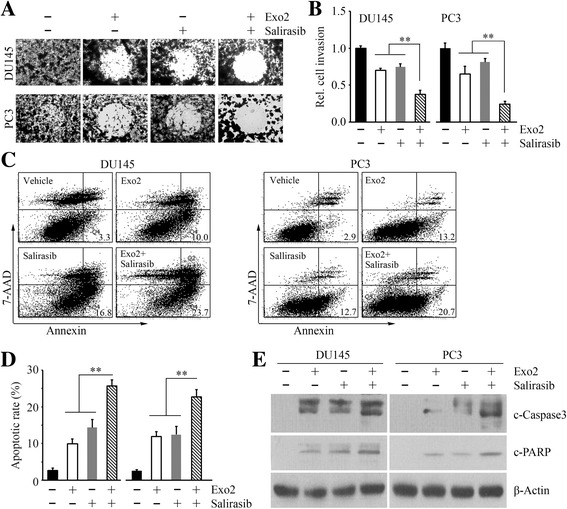
Combination of salirasib and Exo2 suppress migration, invasion and apoptosis of prostate cancer cells more efficiently than either drug alone. **a** DU145 and PC3 cells were treated with 50 μM Exo2 and 50 μM salirasib for 16 h, alone or in combination, and cell migration was determined by gap closure. **b**–**d** DU145 and PC3 cells were treated with 50 μM Exo2 and 50 μM salirasib for 24 h, alone or in combination. Cell invasion was determined by Boyden chamber (**b**), and cell apoptosis was determined by FITC Annexin V Apoptosis Detection Kit I (**c**). For quantification of invasion, the matrigel membranes that contained invading cells were dissolved in 10% acetic acid and read colorimetrically at 590 nm (**b**). Representative images of apoptosis assays are shown in (**c**) and quantitative data are shown in (**d**). **e** DU145 and PC3 cells were treated with 50 μM Exo2 and 50 μM salirasib for 24 h, alone or in combination, and cell lysates were collected for Western blot with the indicated antibodies. **p < 0.01

To address the question whether Exo2 or/and salirasib affect other phenotypes of prostate cancer cells, we treated DU145 and PC3 cells with Exo2, salirasib or their combination and determined cell migration, invasion and apoptosis in response to the different treatments. Gap closure migration and Transwell invasion assays showed that either Exo2 or salirasib inhibited cell migration and invasion, and the inhibitory effects of co-treatment were much greater compared with the single drug effects (Fig. 3a and b). Additionally, both Exo2 and salirasib induced apoptosis in prostate cancer cells and co-treatment led to a higher apoptotic rate (Fig. 3c and d). Western blot showed that salirasib enhanced Exo2-mediated induction of cleaved Caspase 3 and PARP (Fig. 3e). These observations indicate that the combination of Exo2 and salirasib is more potent in suppressing migration, invasion and promoting apoptosis in prostate cancer cells than either drug alone.

### Exo2 inhibits prostate tumor growth in a xenograft mouse model

**Fig. 4:**
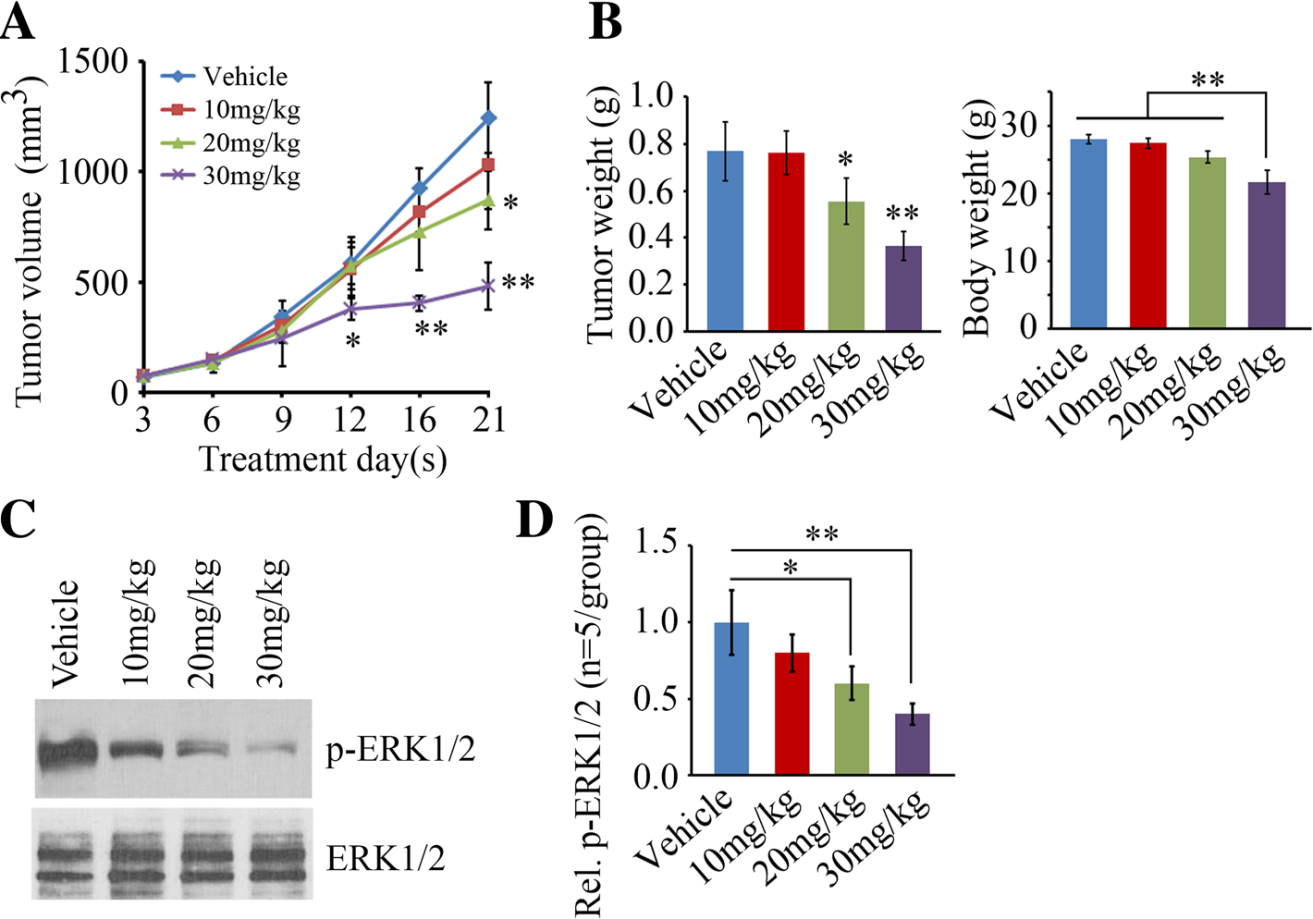
Exo2 exhibits inhibitory activity of prostate tumor growth in vivo. **a**, **b** When PC3-derived xenografts had been established, the SCID mice were randomly divided into four groups for treatment with vehicle or the indicated concentrations of Exo2 (*n* = 5/group). Tumor growth was measured by tumor volume (**a**), tumor weight and mouse body weight (minus tumor) at the end of the experiment was calculated (**b**). **c**, **d** The xenografts were removed these mice for Western blot with the indicated antibodies. Representative result of Western blot is shown in (**c**) and quantitative data are shown in (**d**). **p < 0.01

To assess the impact of Exo2 on prostate tumor growth in vivo, subcutaneous prostate tumor xenografts were established by injecting PC3 cells into NOD/SCID mice. When the xenografts were established, animals were randomly assigned to treatment or control groups. For drug treatment, Exo2 at different dosages (10 mg/kg, 20 mg/kg or 30 mg/kg) was administered to tumor-bearing mice for a total of 3 weeks. A significant reduction of prostate tumor burden was revealed as smaller tumor volume and lower tumor weight following treatment with 20 mg/kg or 30 mg/kg Exo2, compared with the control group (Fig. 4a and b). The tumor burden was not altered when mice were treated with 10 mg/kg Exo2 (Fig. 4a and b). Treatment with 30 mg/kg Exo2 led to a significant body weight loss of mice (Fig. 4b). Indeed, mice treated at the lower, but effective, dose of Exo2 (20 mg/kg) had less average weight loss than those treated with the higher dose (30 mg/kg) and it was tolerated over the duration of multiple treatments (Fig. 4b). These observations suggest that Exo2 dosage higher than 30 mg/kg can induce toxicity which may cause lethality. We collected the xenografts from the different treatment groups and performed Western blot to determine the activation status of MAPK signaling, which showed that phosphorylation levels of ERK1/2 were reduced in the Exo2 treatment group (Fig. 4c and d). Consistent with in vitro data, Exo2 inhibited ERK1/2 activation in a dose-dependent fashion (Fig. 4c and d). Taken together, these findings indicate that Exo2 exhibits anticancer activity by inhibiting the MAPK pathway and the effective dose of Exo2 is 20 mg/kg in SCID mice.

### Combination of Exo2 and salirasib strongly inhibits prostate tumor outgrowth in vivo

**Fig. 5:**
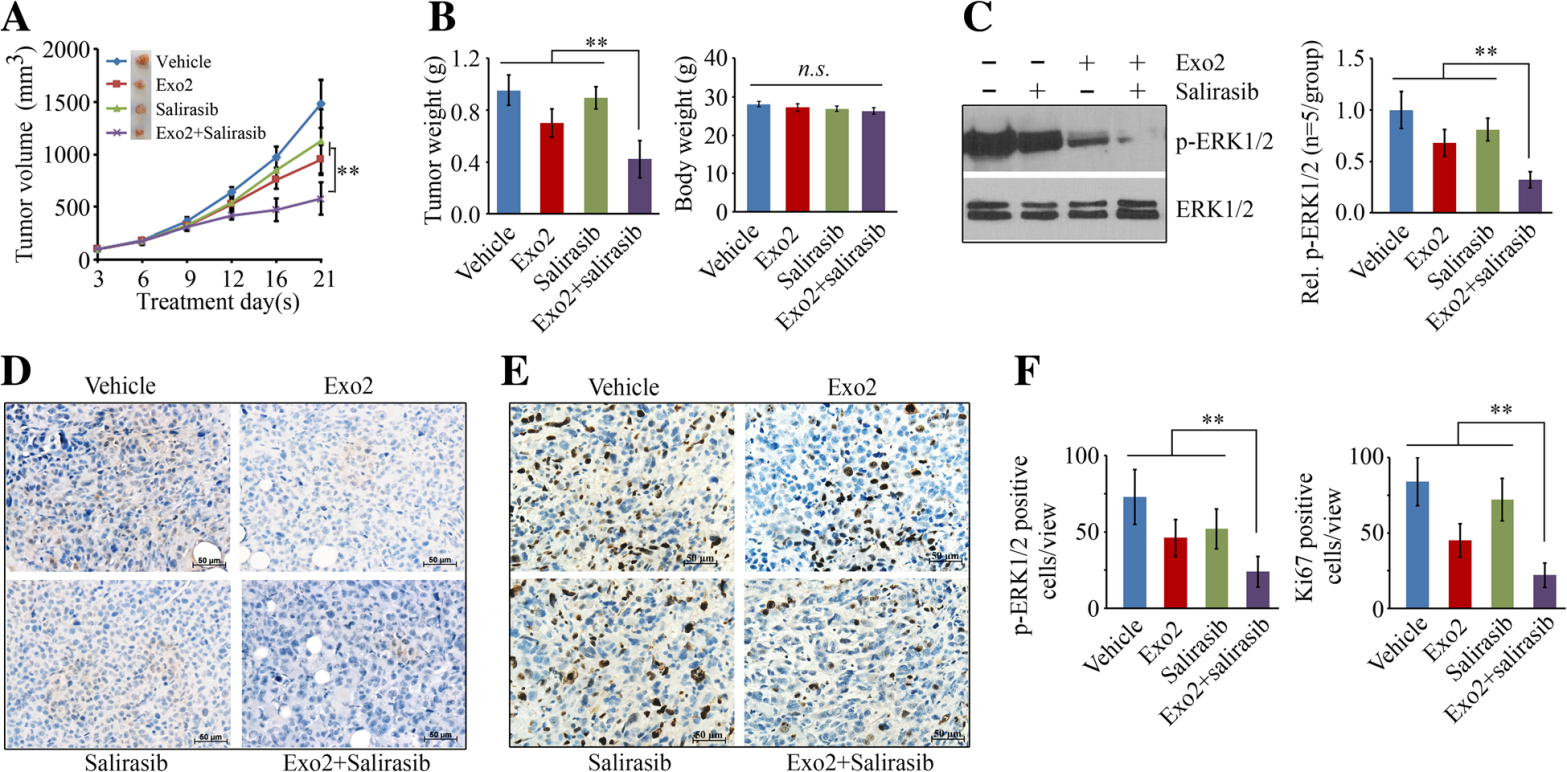
Combination of Salirasib and Exo2 inhibits prostate tumor growth more efficiently than either treatment alone. **a**, **b** When PC3-derived xenografts had been established, the SCID mice were randomly divided into four groups for treatment with vehicle, Exo2, salirasib or the combination of Exo2 and salirasib (*n* = 5/group). Tumor growth was measured by tumor volume and size every 3–5 days (**a**), and tumor and mouse body weight (**b**) was calculated at the end of the experiment. **c** The xenografts were removed from these mice for Western blot with the indicated antibodies. Representative result of Western blot is shown in the left panel and quantitative data are shown in the right panel. **d**–**f** The xenografts removed from the drug-treated tumor-bearing mice were processed for IHC with the indicated antibodies. Representative images of IHC are shown in (**d**, **e**) and quantitative data are shown in (**f**). **p < 0.01

Based on these encouraging data, we generated the SCID-xenograft model to evaluate the synergistic effects of Exo2 (20 mg/kg) and salirasib (20 mg/kg) on prostate tumor growth. After a 3-week treatment, tumor burden was significantly reduced in the Exo2 arm, but not in the salirasib arm (Fig. 5a and b). Although dosage optimization of salirasib treatment in the mouse model still needs to be established, these data indicate that treatment of mice with 20 mg/kg salirasib alone cannot inhibit prostate tumor growth. The reduction in tumor growth was more significant in the mice receiving combination treatment compared with those receiving Exo2 alone (Fig. 5a and b). No significant body weight loss was observed in all these treatments (Fig. 5b). To determine whether MAPK signaling was involved in this synergistic treatment, we examined ERK1/2 activation using the cell lysates collected from the xenografts treated with Exo2 and salirasib, alone or in combination. Western blot analysis showed that either Exo2 or salirasib blocked ERK1/2 activation in prostate tumor cells (Fig. 5c). However, much lower phosphorylation levels of ERK1/2 were found in the dual-treated group compared to single drug-treated groups (Fig. 5c). IHC analysis with phospho-ERK1/2 antibody confirmed that salirasib augmented Exo2-induced repression of ERK1/2 activation (Fig. 5d and f). IHC staining also showed that treatment with Exo2 in the presence of salirasib can significantly reduce a cell proliferation marker Ki67 (Fig. 5e and f). These data demonstrate that combination of Exo2 and saralisab has a superior inhibitory activity on prostate tumor growth compared to monotherapy.

## Discussion

Prostate cancer is the most common cancer among American men (1). Decades of research to discover efficacious drugs that can block the oncogenic pathways in prostate cancer development and progression have yielded modest achievements [1]. Particularly for the management of advanced prostate cancer, current options are limited, and call for new and more effective treatment approaches. Although the MAPK and PI3K/AKT cascades have been identified as the master signaling pathways controlling the prostate cancer progression, targeting these nodes in the signaling cascades individually typically involves a switch to the other pathway in a rescue strategy by cancer cells to overcome monotherapies [33–35]. Therefore, seeking a way to inhibit PI3K/AKT or MAPK pathway and prevent the switch to alternative usage of these two parallel cascades is indeed important for prostate cancer treatment.

**Fig. 6:**
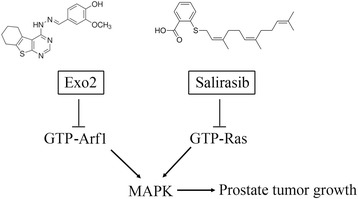
Schematic representation of the mechanism of combination-mediated inhibition of prostate tumor growth

This study aimed to investigate the effect of a combination of Exo2 and salirasib in prostate cancer to achieve better therapeutic benefit with the intention that this approach would eventually reduce single drug toxicity, and perhaps minimize or delay the induction of drug tolerance. In this study, we evaluated the effect of Exo2 alone, and in combination with salirasib in vitro in cultured prostate cancer cell lines and in vivo in a subcutaneous xenograft mouse model. We demonstrate here that the combination of Exo2 and salirasib exhibits a superior anticancer activity in prostate cancer, which is mediated at least in part by suppression of tumor growth through co-inhibition of Arf1- and Ras-mediated MAPK activity in cancer cells (Fig. 6). Critically, the drug effects on suppression of MAPK pathway prevent the switch to PI3K/AKT signaling. Our results suggest that synergistic treatment may represent a more efficacious therapeutic regimen for eradicating prostate cancer.

The expression of the androgen receptor gene in prostate cancer cells is regulated by androgens, and both androgen receptor (AR) and growth factor pathways can trigger ERK MAPK signaling in prostate cancer cells [36, 37]. To determine whether the efficacy of Exo2 depends on AR activity, we utilized four prostate cancer cell lines in this study. DU145, PC3 and 22Rv1 are androgen-independent prostate cancer cells, while LNCaP is a well-characterized cell model for androgen-dependent. Our data explored that Exo2 can suppress ERK1/2 activation and proliferation regardless of the presence of AR on the cell, suggesting that Exo2 may have broad anticancer effects on prostate cancer.

Ras mutations have been implicated in the pathogenesis of a variety of human cancers. Although Ras is rarely mutated in prostate cancer, its activation has been associated with the grade of prostate cancer [10, 38]. To seek the link between the Ras gene status and cell sensitivity to the Ras inhibitor salirasib, we utilized various cell lines with different Ras genetic backgrounds. MDA-MB-231, H1299 and SW60 cells carry Ras mutations, and other cell lines examined in this study have wild-type Ras. At the same concentration of salirasib (within 50 μM), cancer cells harboring Ras mutations did not show increased sensitivity to salirasib compared with cancer cells with wild-type Ras. Moreover, salirasib treatment reduced phosphorylation of ERK1/2 in cultured prostate cancer cells and prostate tumor xenografts. This study indicates that salirasib acts independently of Ras mutations, which may provide a rationale for testing it in the treatment of prostate cancer. Although salirasib has shown great anticancer potential by selectively inhibiting the growth of cells with activated Ras, salirasib monotherapy was unsuccessful in a phase II clinical trial [15, 39]. To achieve adequate outcomes, salirasib-based combination therapy with the addition of Arf1 inhibitors opens up a new avenue to combat prostate cancer.

Anticancer activity produced by the combination of Exo2 and salirasib is associated with inhibition of migration/invasion and promotion of apoptosis. ERK MAPK signaling appears to play a major role in proliferation, survival and differentiation [25, 36]; therefore, other pathways may be involved in mediating drug action. In this study, we also investigated the Src and STAT3 pathways, and there were no obvious changes in their activation when compared with combination and single treatment (data not shown). Nevertheless, more efforts need to be made to elucidate the mechanisms underlying combination-mediated inhibitory effects, particularly on prostate cancer bone metastasis.

Recently, novel molecules have been developed to overcome the disadvantages of existing Arf1 inhibitors. Those includes AMF26, LM11 and their derivatives [40, 41]. We previously reported that a small molecule LM11 can effectively impair Arf1 activation in breast cancer cells and suppress their metastatic capability in zebrafish [21]. LM11 impairs Arf1 activation more strongly than Exo2 in cancer cells [21]; therefore, our follow-up investigations will be focused on studying LM11 efficacy and its potential synergistic effects with salirasib in prostate cancer.

## Conclusions

This study bridges two important small GTPases Ras and Arf1 with an emphasis on ERK MAPK signaling in the development of prostate cancer. Assessment of Arf1 inhibitor efficacy in a preclinical model and evaluation the synergistic anticancer effects of the combination of Arf1 and Ras inhibitors, have immediate potential in reducing cancer morbidity and improving the quality of life of those affected by prostate cancer. Additionally, this novel approach to combat prostate cancer by simultaneous blockade of Arf1- and Ras-mediated signaling cascades, has significant impact on the design and execution of effective therapy for prostate cancer patients.

## Additional file

Additional file 1:
**Figure S1.** Prostate cancer cell lines DU145, PC3, 22Rv1 and LNCaP were treated with the indicated concentrations of Exo2 for 1 week, and cell proliferation was determined by crystal violet staining. In these assays, the dye was dissolved in 1% SDS after staining with 0.5% crystal violet and measured at 570 nm (OD570) with a plate reader. Representative images and quantitative data are shown in (A) and (B), respectively. **p* < 0.05; ***p* < 0.01. (TIFF 2086 kb)

## Abbreviations

ADP-ribosylation factors: Arfs
ADT: Androgen deprivation therapy
AR: Androgen receptor
Arf1 GAPs: Arf1 GTPase-activating proteins
Arf1 GEFs: Arf1-directed guanine nucleotide exchange factors
FTS: Farnesylthiosalicylate
MAPK: Mitogen-activated protein kinase
mCRPC: Metastatic castration-resistant prostate cancer
PI3K: Phosphoinositide-3-kinase
