# Supplementary figures and images for: Combined targeting of Arf1 and Ras potentiates anticancer activity for prostate cancer therapeutics
Source: J Exp Clin Cancer Res. 2017 Aug 23;36:112. doi: 10.1186/s13046-017-0583-4 (PMC5568197; doi:10.1186/s13046-017-0583-4)

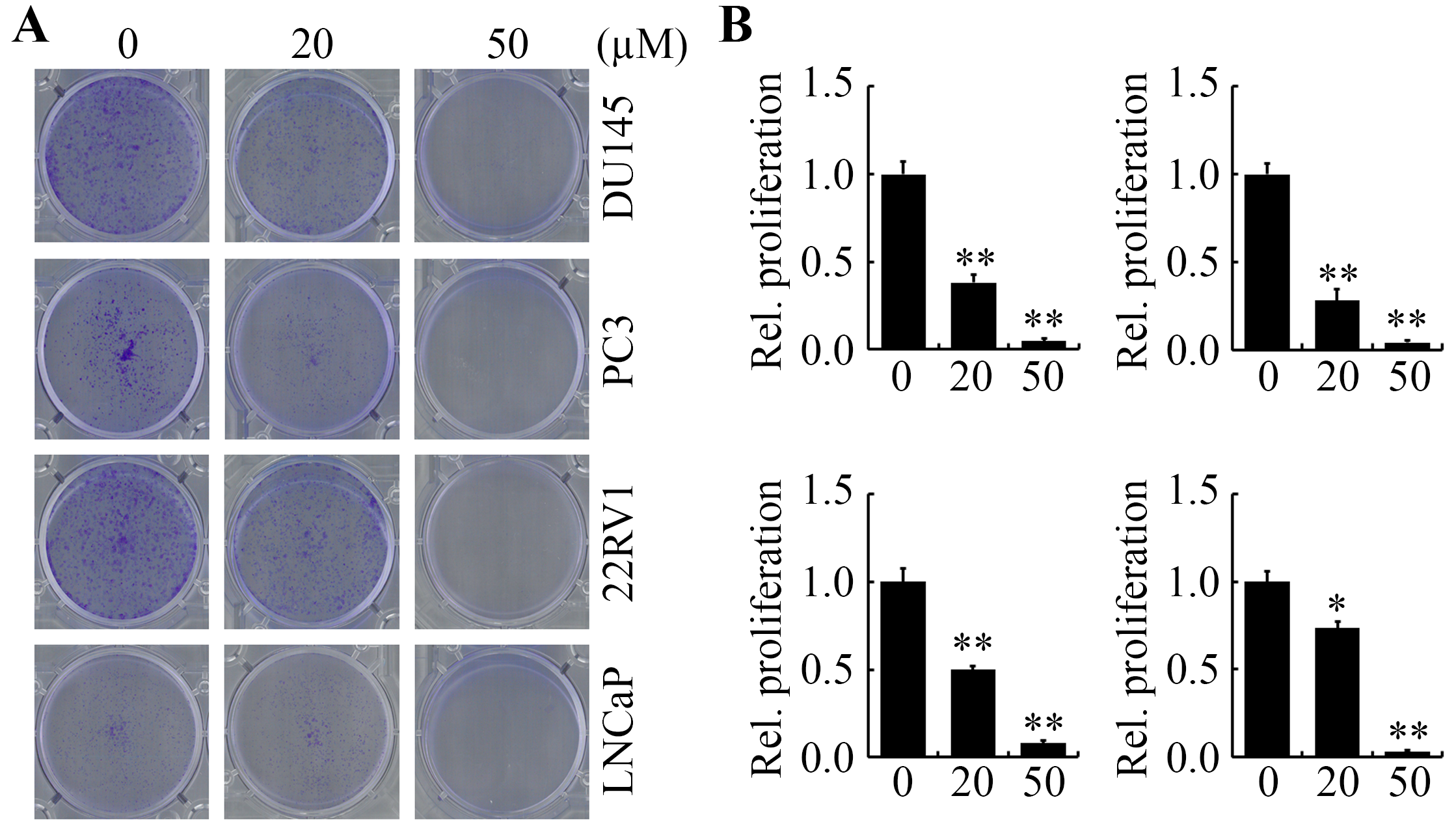

Supplement: Additional file 1: — Figure S1. Prostate cancer cell lines DU145, PC3, 22Rv1 and LNCaP were treated with the indicated concentrations of Exo2 for 1 week, and cell proliferation was determined by crystal violet staining. In these assays, the dye was dissolved in 1% SDS after staining with 0.5% crystal violet and measured at 570 nm (OD570) with a plate reader. Representative images and quantitative data are shown in (A) and (B), respectively. *p < 0.05; **p < 0.01. (TIFF 2086 kb) [file 13046_2017_583_MOESM1_ESM.tif]
